# Genome-wide insights of shared genetic architecture between menstrual traits and bone mineral density

**DOI:** 10.1530/EC-25-0278

**Published:** 2025-10-23

**Authors:** Bowen Lei, Lin He, Yang Qu, Xueyao Wu, Lingli Qiu, Mingshuang Tang, Li Zhang, Yanqiu Zou, Xin Song, Bin Yang, Wenzhi Wang, Lei Sun, Lu Wang, Jian Xu, Yao Chen, Shouzhong Wang, Shu Cai, Mengyu Fan, Jiayuan Li, Ben Zhang, Xia Jiang, Yiping Jia

**Affiliations:** ^1^ Department of Epidemiology and Biostatistics, Institute of Systems Epidemiology, West China School of Public Health and West China Fourth Hospital, Sichuan University, Chengdu, Sichuan, China; ^2^ Longquanyi District of Chengdu Maternity and Child Care Health Hospital, Chengdu, Sichuan, China; ^3^ Department of Osteoporosis, West China School of Public Health and West China Fourth Hospital, Sichuan University, Chengdu, Sichuan, China; ^4^ Department of Radiology, West China School of Public Health and West China Fourth Hospital, Sichuan University, Chengdu, Sichuan, China; ^5^ Hainan General Hospital and Hainan Affiliated Hospital, Hainan Medical University, Haikou, China; ^6^ Department of Nutrition and Food Hygiene, West China School of Public Health and West China Fourth Hospital, Sichuan University, Chengdu, Sichuan, China; ^7^ Department of Clinical Neuroscience, Karolinska Institutet, Stockholm, Sweden; ^8^ Department of Ultrasound, West China School of Public Health and West China Fourth Hospital, Sichuan University, Chengdu, Sichuan, China

**Keywords:** age at menarche, age at menopause, osteoporosis, heel estimated bone mineral density, genetic correlation, pleiotropic loci, causal inference

## Abstract

**Objective:**

While the phenotypic associations of menstrual traits, such as age at menarche (AAM) and age at natural menopause (ANM), with bone mineral density (BMD) have been well observed, the understanding of their shared genetic mechanisms is lacking. We aimed to systematically explore the underlying genetic basis connecting AAM and ANM with BMD.

**Methods:**

We performed a large-scale genome-wide cross-trait analysis by leveraging summary statistics from the hitherto largest genome-wide association studies conducted among the European population for AAM (*n* = 556,124), ANM (*n* = 201,323), and heel estimated BMD (eBMD, *n* = 426,824), a robust validated predictor of osteoporosis risk.

**Results:**

We identified significant genetic correlations for eBMD with both AAM (*r*_g_ = −0.082, *P* = 1.83 × 10^−8^) and ANM (*r*_g_ = 0.044, *P* = 0.007). Cross-trait meta-analysis yielded 203 AAM-eBMD shared loci, of which 3 were novel and 77 ANM-eBMD shared loci, of which two were novel. Gene-based analysis revealed 409 AAM-eBMD shared genes and 179 ANM-eBMD shared genes. Mendelian randomization demonstrated that genetically predicted later AAM (*β* = −0.054, 95% CI = −0.069 to −0.040, *P* = 6.08 × 10^−14^) and genetically predicted earlier ANM (*β* = 0.010, 95% CI = 0.004–0.017, *P* = 0.003) were significantly associated with decreased levels of eBMD. No evidence of reverse causality was found.

**Conclusion:**

Our work provides evidence in support of a substantial shared genetic basis and causal relationships between menstrual traits and BMD. The findings could be instrumental in developing risk stratification strategies and formulating novel pharmaceutical interventions for osteoporosis.

**Strengths and limitations of the study:**

- Genome-wide cross-trait design provided insights into the shared genetic basis and biological mechanisms underlying the phenotypic associations between AAM, ANM, and eBMD.
- The adoption of the hitherto largest GWAS summary statistics ensured statistical power.
- The genetic data were exclusively from European ancestry, limiting the generalizability of our results.
- Current findings are primarily derived from bioinformatic analyses and interpreted based on existing, incomplete biological knowledge of the SNPs and genes.

## Introduction

Age at menarche (AAM) and age at natural menopause (ANM) represent critical reproductive milestones that have been implicated in bone health through observational studies. Later AAM has been associated with decreased bone mineral density (BMD) (1, 2), a reliable indicator of osteoporosis risk, while later ANM has been linked to increased BMD (3, 4). Conventional observational studies, however, are susceptible to biases, such as unmeasured confounding and reverse causality, warranting the application of alternative study designs to strengthen causal inference (5). Moreover, although the underlying mechanisms driving these associations are likely multifactorial, substantial emphasis has been placed on the potential role of estrogen exposure (1, 3, 6), while the involvement of other biological pathways remains to be fully elucidated.

Genetic variants, which can be mapped to biological pathways to elucidate mechanisms connecting interrelated traits, are randomly assigned at conception and remain fixed throughout life. Therefore, leveraging genetic approaches to explore relationships between AAM, ANM, and BMD not only mitigates confounding and reverse causality concerns but also provides novel insights into the underlying biology linking these interconnected traits. Utilizing genome-wide single nucleotide polymorphism (SNP) information (7), Trajanoska *et al.* reported negative (although nonsignificant) genetic correlations between AAM and BMD across different sites (femoral neck (FN) *r*_g_ = −0.08, lumbar spine (LS) *r*_g_ = −0.06) and positive genetic correlations for ANM (FN *r*_g_ = 0.05, LS *r*_g_ = 0.04) (8). Such results suggest a non-negligible shared genetic basis, which can be attributed to pleiotropy (i.e., specific genetic components are involved in both traits) or/and a causal relationship. Four previous Mendelian randomization (MR) studies provided further evidence on a negative causal effect of AAM on BMD (8, 9, 10) and a positive causal effect of ANM on BMD (8, 11).

Despite these advancements, several key issues remain to be addressed. First, a systematic investigation into the specific genetic components accounting for the observed genetic correlations is absent. By comparing the results of GWAS that examined menstrual traits and BMD separately, multiple loci can be immediately recognized as co-regulating AAM and BMD (e.g., *RERE*, *CTPS1*, and *NEGR1*) (12, 13) or ANM and BMD (e.g., *DMC1* and *MSH6*) (11, 13, 14), indicating the potential to discover additional yet unrevealed common loci with improved statistical power through combining datasets across traits. Second, recent large-scale GWAS for menstrual traits and BMD have identified a greater number of genetic variants with more robustly estimated effects, attributable to the substantial expansion of sample size. This underscores the necessity of updating prior MR studies with enhanced accuracy. Third, the univariable design of previous MR studies neglected the inclusion of common confounders (e.g., physical activity and smoking (15)), which might have violated the instrument independence assumption (16), leading to spurious association signals. Finally, no prior studies have investigated whether BMD or osteoporosis might affect reproductive lifespan in women.

Therefore, the current study aimed to perform a comprehensive genome-wide cross-trait analysis that can expand existing MR findings, as well as interrogate the common genetic influences underlying phenotypic correlations observed for AAM and ANM with BMD. To this aim, leveraging by far the largest genetic variation data of each trait, we aimed to: i) examine genome-wide and local genetic correlations, ii) identify specific shared genetic loci, genes, and biological pathways, and iii) assess the bi-directional causal relationships between each of the two menstrual traits and BMD.

## Materials and methods

**Figure 1:**
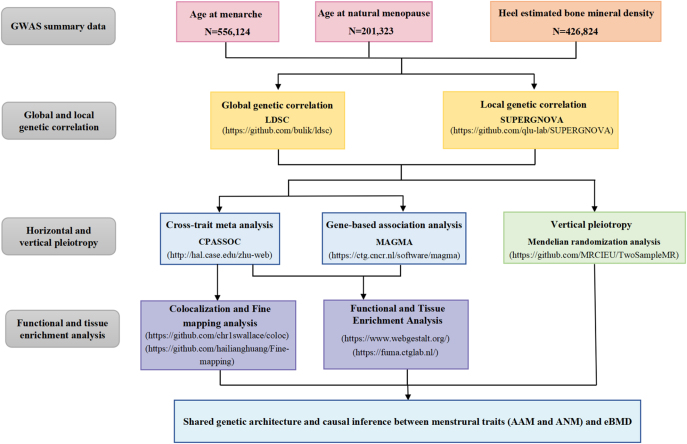
Flow chart of the overall study design. GWAS summary data for menstrual traits (AAM and ANM) and eBMD were retrieved. We first quantified the global and local genetic correlation between menstrual traits and eBMD. Then we applied cross-trait meta-analysis and gene-based association analysis to identify pleiotropic loci, and further carried out functional analysis to understand the potential biological mechanisms. Finally, a Mendelian randomization analysis was conducted to make causal inference. AAM, age at menarche; ANM, age at natural menopause; eBMD, heel estimated bone mineral density.

Our overall study design is presented in Fig. 1.

### GWAS datasets

#### Menstrual traits

For AAM, we obtained GWAS summary data from a meta-analysis of the ReproGen Consortium, the Breast Cancer Association Consortium, the Ovarian Cancer Association Consortium, UK Biobank (UKB), and 23andMe, consisting of up to 632,955 women of European ancestry (17). For ANM, we obtained GWAS summary data from a meta-analysis of 1,000 Genomes-imputed studies, the Breast Cancer Association Consortium (BCAC), and the UKB, consisting of 201,323 women of European ancestry (11). The two menstrual traits were both assessed in years through specific questions (e.g., ‘how old were you when your menstruation started?’ for AAM and ‘age when menstrual periods permanently stopped’ for ANM).

We extracted the effect size and relevant information of the 901 and 227 (*P* < 5 × 10^−8^) genome-wide significant and independent autosomal SNPs for AAM and ANM, and used those SNPs as instrumental variables (IVs) in MR analyses. We also downloaded the full set of summary statistics for AAM, excluding the 23andMe component due to limited data availability, as well as the full set of summary statistics for ANM.

#### Estimated bone mineral density

We adopted summary-level data for heel estimated BMD (eBMD) from the largest available GWAS conducted by Morris and colleagues (13), including 426,824 European individuals (55% female) from UKB. eBMD was assessed by measuring quantitative heel ultrasound and measured in g/cm^3^. This method is quick, safe, and relatively inexpensive and can therefore be assessed in very large sample sizes. We adopted the genome-wide significant *P*-threshold of 6.6 × 10^−9^ as defined in the original GWAS, yielding 1,103 independent signals as IVs for bidirectional MR analysis. Detailed characteristics of each included GWAS can be found in Supplementary Table S1 (see section on Supplementary materials given at the end of the article).

### Patient and public involvement

Patients and the public were not involved in the design, conduct, reporting, or dissemination plans of this research.

### Statistical analyses

#### Global genetic correlation analysis

We employed the LDSC method (7) to evaluate the global genetic correlation, which quantifies the overall shared genetic basis between menstrual traits and eBMD. LDSC estimates genetic similarity between two traits by examining whether genetic variants that increase risk for one trait also tend to increase or decrease risk for another trait across the genome, achieved through analyzing how the genetic association statistics (Z-scores) from two different GWAS studies correlate. It accounts for the regional correlation pattern among SNPs, known as linkage disequilibrium (LD), the tendency for genetic variants located close together on a chromosome to be inherited together – which can otherwise lead to spurious associations. The estimated genetic correlation (*r*_g_) ranges from −1 to +1, with −1 indicating a perfect negative correlation and +1 indicating a perfect positive correlation. We used pre-calculated HapMap3 LD scores computed from ∼1.2 million common SNPs in European ancestry, regarded as well-imputed in most studies. A Bonferroni-corrected *P*-threshold of 0.05/2 (number of menstrual traits) was used to define statistical significance.

#### Local genetic correlation analysis

We further estimated local genetic correlation using SUPERGNOVA (18), which identifies specific genomic regions (local areas of chromosomes) that contribute most to the genetic similarity between two traits. Instead of estimating the average genetic correlation across the genome, SUPERGNOVA pre-partitions the genome into 2,353 approximately LD-independent regions with an average length of 1.6 centimorgans, decorrelates Z-scores of SNPs within each region using eigenvector decomposition, and calculates genetic correlation in these specific regions. A Bonferroni-corrected *P*-threshold of 0.05/2,353 (number of genomic regions) was used to define statistical significance.

#### Cross-trait meta-analysis

We next performed a cross-trait meta-analysis by utilizing cross-phenotype association (CPASSOC) to identify pleiotropic loci influencing both traits (19). CPASSOC integrates summary-level GWAS data and analyzes multiple traits simultaneously with improved statistical power, accounting for overlapping or related samples and cryptic relatedness. The test statistic S_Het_ maintains statistical power even under population heterogeneity due to the use of summary statistics from multiple cohorts, and was adopted in this analysis.

We applied PLINK’s clumping function to obtain independent shared loci (parameters: – clump-p1 5e-8 – clump-p2 1e-5 – clump-r2 0.05 – clump-kb 500) (20). SNPs with the lowest *P*-value within each independent locus were selected as index SNPs. SNPs with *P*_CPASSOC_ < 5 × 10^−8^ and *P*_single-trait_ < 1.0 × 10^−5^ were identified as significant pleiotropic SNPs. Specifically, these SNPs were further categorized into four groups: i) ‘known’ shared SNPs, i.e., SNPs that reached genome-wide significance in both traits; ii) ‘single-trait-driven’ shared SNPs, i.e., SNPs reached genome-wide significance in only one of the two traits; iii) ‘LD-tagged’ shared SNPs that, despite not being driven by a single trait, were in LD (*r*^2^ > 0.20 or within one MB region) with previously reported significant SNPs in single-trait GWAS; iv) ‘novel’ shared SNPs, which we mainly focused on, were SNPs neither driven by any single trait nor in LD with index SNPs identified by single-trait GWAS.

We employed Ensembl variant effect predictor (VEP) and 3DSNP for detailed functional annotation of the identified pleiotropic SNPs. VEP identifies candidate genes by examining which genes are located nearest to each SNP on the linear chromosome map, providing straightforward SNP-gene associations based on genomic proximity (21). 3DSNP incorporates three-dimensional chromatin structure data to identify genes that may be regulated by SNPs through chromosomal folding and looping mechanisms (22).

#### Colocalization analysis

To determine whether two traits share the same causal genetic variant or simply have associated variants in the same chromosomal region, we performed a colocalization analysis using Coloc (23). This method provided posterior probabilities for five mutually exclusive hypotheses regarding the sharing of causal variants in a genetic region, including i) H0: no association; ii) H1: association with trait 1 only; iii) H2: association with trait 2 only; iv) H3: association with both traits but two distinct SNPs; and v) H4: association with both traits and one shared SNP. We extracted summary statistics for variants within 500 kb of the index SNP at each shared locus and calculated the posterior probability for H4 (PPH4). A locus was considered colocalized if PPH4 was greater than 0.8.

#### Fine mapping credible set analysis

Given that the index SNP showing the strongest association is often not the actual causal SNP responsible for the biological effect, but rather a correlated marker linked to the true causal SNP through linkage disequilibrium, we performed fine mapping analysis using FM-summary to identify credible sets of variants with 99% probability of containing the causal variants at each pleiotropic locus (24). This algorithm maps the primary signal and uses a flat prior with the steepest descent approximation, assuming at least one causal variant exists within a given region.

#### Gene-based analysis

SNP-based approaches mostly suffer from enormous multiple testing burdens, which limit statistical power, and tend to miss weaker associations. Given this, we continued to conduct a gene-based analysis by using MAGMA (25). MAGMA aggregates genetic marker data to the level of genes, and tests the joint association of all markers in each gene with a certain trait, relying on a multiple linear principal component regression model. We first identified an independent set of significantly associated genes for each trait, and then intersected AAM- or ANM-associated gene sets with the eBMD-associated gene set to obtain shared genes.

#### Pathway and tissue-specific enrichment analysis

To provide biological insights into the pleiotropic loci, we further performed pathway and tissue-specific enrichment analysis using CPASSOC-identified genes annotated by VEP and 3DSNP, as well as shared genes identified by MAGMA. We applied the WebGestalt (26) tool to assess the enrichment of shared genes in GO biological processes or KEGG pathways. We also conducted GTEx tissue enrichment analysis using the FUMA GENE2FUN process (27). The Benjamini–Hochberg procedure was used to correct for multiple testing (false discovery rate <0.05).

#### Mendelian randomization analysis

To investigate whether menstrual traits have causal effects on eBMD rather than association, we conducted comprehensive two-sample MR analyses using genetic variants as IVs, with AAM and ANM serving as exposures. The proportion of variance explained and *F*-statistics were used to measure the strength of IVs. The random-effect inverse-variance weighted (IVW) method (28) was used as the primary approach, complemented by the weighted median method (29) and MR-Egger regression (30). The effect sizes were expressed as beta coefficients representing the standard deviation (SD) change in eBMD per additional year of AAM or ANM. In addition, we conducted a series of sensitivity analyses to validate the MR results, including: i) exclusion of palindromic IVs with strand ambiguity; ii) exclusion of pleiotropic IVs (associated with outcomes or potential confounders) confirmed by GWAS Catalog screening and by CPASSOC and MAGMA analyses performed earlier in this study; iii) leave-one-out analysis to examine if any single SNP drove the association; iv) MR-Pleiotropic Residual Sum and Outlier (MR-PRESSO) (31) to detect horizontal pleiotropy and re-calculate the effects after removing outliers; v) sensitivity analysis utilizing GWAS summary statistics from non-overlapping samples. The multi-variable MR (MVMR) (16) method was also employed to estimate the direct unconfounded effect of menstrual traits on eBMD, controlling for genetic predisposition to body mass index (BMI) (32), lifetime smoking index (33), physical activity (34), educational attainment (EA) (35), and ANM/AAM. To rule out reverse causality, we performed reverse-direction MR analyses using eBMD as the exposure, with results presented as per-year change in AAM or ANM per SD increase in eBMD.

#### Supplementary analysis

To minimize the influence of sex heterogeneity, we replaced our sex-combined eBMD GWAS with a female-specific eBMD GWAS including 111,152 women, a quarter of the sample size of the sex-combined GWAS. We then re-ran the analytical pipeline to validate our primary results, ensuring the robustness of our findings.

## Results

### Global and local genetic correlation

Using LDSC, we estimated the SNP-based heritability of AAM, ANM, and eBMD to be 20.84, 19.20, and 38.05%, respectively. We observed a modest negative AAM-eBMD global genetic correlation (*r*_g_ = −0.082, *P* = 1.83 × 10^−8^), indicating that variants associated with earlier AAM tend to increase eBMD levels. Conversely, the positive ANM-eBMD global genetic correlation (*r*_g_ = 0.044, *P* = 0.007) shows that variants associated with earlier ANM tend to decrease eBMD levels. In addition, a significant positive genetic correlation between AAM and ANM was observed (*r*_g_ = 0.050, *P* = 0.001).

**Figure 2:**
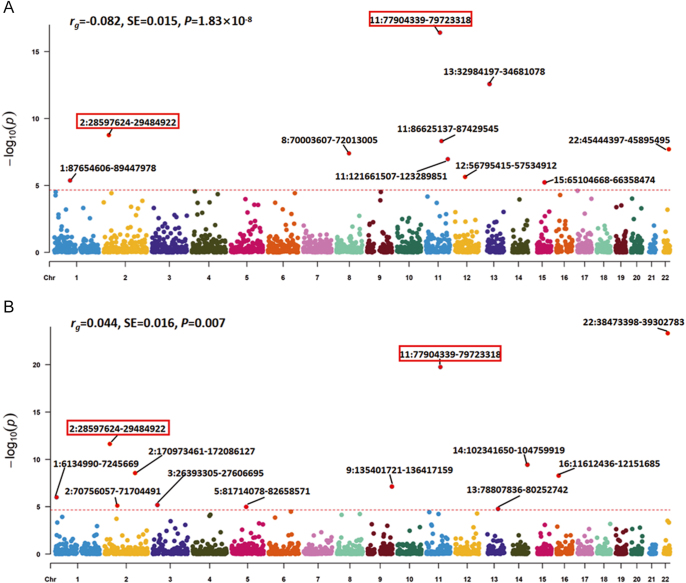
Global and local genetic correlations between menstrual traits and eBMD. (A) Manhattan-style plot of genetic correlation between AAM and eBMD with global genetic correlation obtained by LDSC shown on the upper left. (B) Manhattan-style plot of local genetic correlation between ANM and eBMD with global genetic correlation obtained by LDSC shown on the upper left. Red dashed lines denote the significance threshold after multiple testing correction (0.05/2,353), red dots represent independent genetic regions showing significant local genetic correlation, and red rectangles mark genetic regions shared in (A) and (B). AAM, age at menarche; ANM, age at natural menopause; eBMD, heel estimated bone mineral density; rg, genetic correlation coefficient; SE, standard error.

Dividing the whole genome into 2,353 LD-independent regions, we further identified ten genetic regions exhibiting a significant local correlation for AAM and eBMD (Fig. 2A). The most significant region was located at 11q14.1 (chr11: 77,904,339–79,723,318, *P* = 2.01 × 10^−18^), harboring *GAB2*, associated with AAM (36, 37) and ANM (38), and *USP35*, associated with AAM (39). Moreover, 12 significant shared genetic regions were identified for ANM and eBMD (Fig. 2B). Notably, four genetic regions (1p36.31, 2p23.2, 5q14.1-q14.2 and 11q14.1), harboring previously reported eBMD loci (e.g., *PHF13*, *PLB1*, and *ATP6AP1L*) and ANM loci (e.g., *DNAJC11* and *GAB2*), were jointly identified as shared regions in both AAM-eBMD and ANM-eBMD analyses. Detailed information on local genetic correlations is presented in Supplementary Tables S2 and S3.

### Cross-trait meta-analysis and colocalization

**Figure 3:**
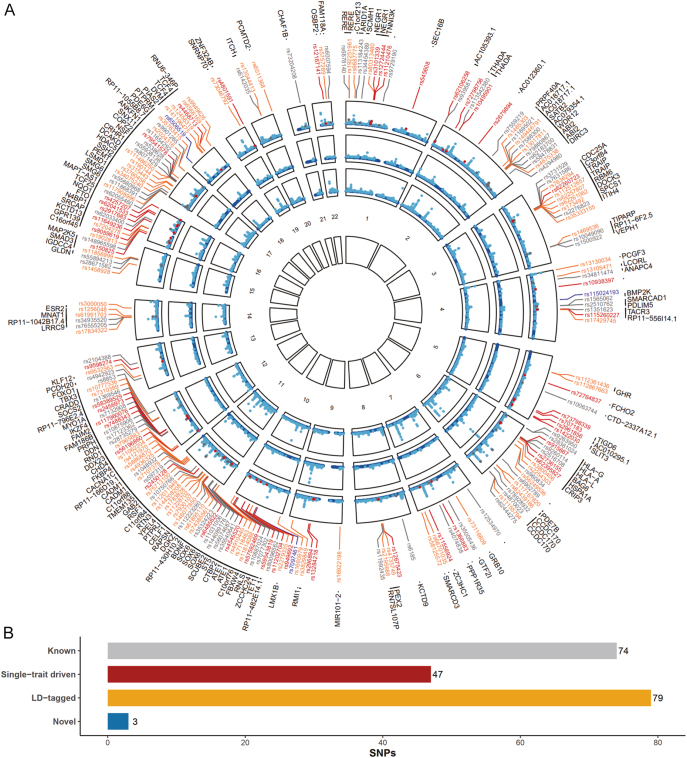
Pleiotropic loci between AAM and eBMD identified by CPASSOC. (A) In the circular Manhattan plot, the outermost circle shows the cross-phenotype association (CPASSOC) results between AAM and eBMD; from periphery to center, each circle shows the GWAS results for AAM and eBMD, respectively. The wathet blue indicates variants with genome-wide significance (*P*_AAM_ < 5.0 × 10^−8^ or *P*_eBMD_ < 6.6 × 10^−9^), whereas the dark blue indicates variants whose *P*-value did not reach the genome-wide significance threshold. A ‘known’ shared SNP is a SNP reaching genome-wide significance in both single-trait GWAS (*P*_AAM_ < 5.0 × 10^−8^ and *P*_eBMD_ < 6.6 × 10^−9^) and expectedly with *P*_CPASSOC_ < 5.0 × 10^−8^; A ‘single-trait-driven’ shared SNP is a SNP reaching genome-wide significance in only one single-trait GWAS (*P*_AAM_ < 5.0 × 10^−8^ or *P*_eBMD_ < 6.6 × 10^−9^) and with *P*_CPASSOC_ < 5.0 × 10^−8^; a ‘LD-tagged’ shared SNP is a SNP not reaching genome-wide significance in either single-trait GWAS (*P*_AAM_ > 5.0 × 10^−8^ and *P*_eBMD_ > 6.6 × 10^−9^) and with *P*_CPASSOC_ < 5.0 × 10^−8^, but in LD with index SNPs (or any SNP located within 1.0 Mb around the index SNPs) already identified by single-trait GWAS(s); a ‘novel’ shared SNP is a SNP not reaching genome-wide significance in either single-trait GWASs but *P*_single-trait_ < 5.0 × 10^−5^ and *P*_CPASSOC_ < 5.0 × 10^−8^, and not in LD with previously identified AAM/ANM-associated or eBMD-associated SNPs. These four types of SNPs are presented in gray, dark red, orange, and blue, respectively. RS IDs of the four types of SNPs and their closest mapped genes to pleiotropic loci are listed around the circles. (B) The bar plot presents the numbers of the four types of SNPs detected in the cross-trait meta-analysis between AAM and eBMD. GWAS, genome-wide association analysis; AAM, age at menarche; eBMD, heel estimated bone mineral density; LD, linkage disequilibrium; SNP, single-nucleotide polymorphism.

**Figure 4:**
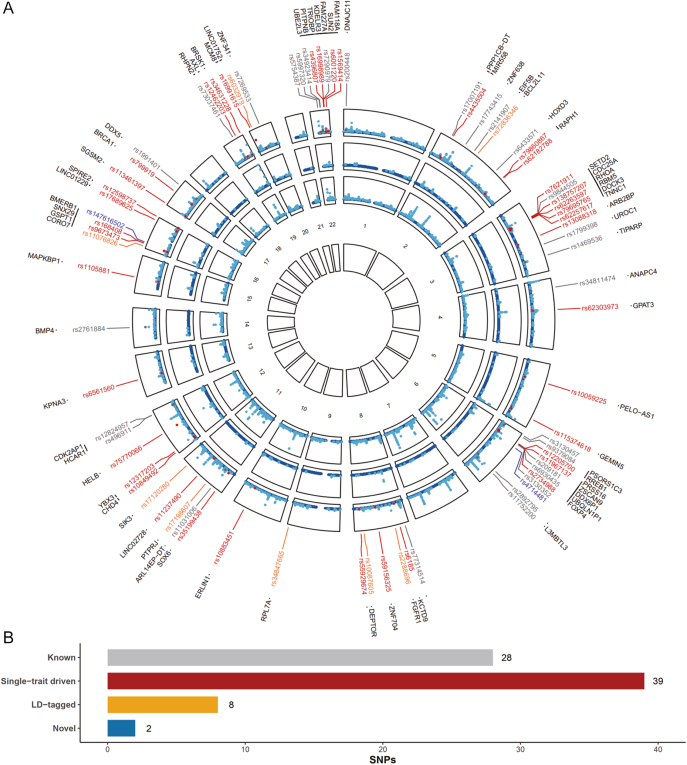
Pleiotropic loci between ANM and eBMD identified by CPASSOC. The (A) circular Manhattan plot and (B) bar plot for ANM-eBMD cross-trait meta-analysis results. The detailed explanation is the same as Fig. 3, except the trait analyzed is ANM instead of AAM. ANM, age at natural menopause; eBMD, heel estimated bone mineral density.

Given the significant genetic overlap observed for menstrual traits and eBMD, we next performed a cross-trait meta-analysis to detect pleiotropic loci. In total, 280 independent loci reached genome-wide significance in CPASSOC (*P*_CPASSOC_ <5 × 10^−8^ and *P*_AAM/ANM_ <1 × 10^−5^ and *P*_eBMD_ <1 × 10^−5^), including 203 AAM-eBMD shared loci and 77 ANM-eBMD shared loci (Figs 3 and 4 and Supplementary Tables S4 and S5). After excluding 203 AAM-eBMD pleiotropic loci, we observed an approximately 15% decrease in SNP-heritability for both AAM and eBMD. After excluding 77 ANM-eBMD pleiotropic loci, we observed an 18% decrease in SNP-heritability for ANM and a 6% decrease for eBMD (Supplementary Fig. S1).

Five loci (index SNPs rs11031006, rs1469536, rs34811474, rs6185, and rs79860867) were shared by both pairs of traits. Among these shared pleiotropic loci, rs34811474 exhibited a reproductive span-decreasing effect (where AAM-increasing alleles coincide with ANM-decreasing alleles) and demonstrated negative effects on eBMD. Two SNPs (rs11031006 and rs1469536) showed right-shift effects (where AAM-increasing alleles also increase menopause age), and two SNPs (rs6185 and rs79860867) exhibited left-shift effects on reproductive span, with all four loci demonstrating varying directional effects on eBMD (Supplementary Table S6).

After excluding SNPs that were in LD (*r*^2^ > 0.20 or within one MB region) with any of the previously reported single-trait-associated significant SNPs, we identified three novel pleiotropic SNPs (rs115024193, rs6506519, and rs7097461) for AAM and eBMD and two novel pleiotropic SNPs (rs4714481 and rs147616502) for ANM and eBMD (Table 1). Among the identified pleiotropic loci, 92 AAM-eBMD loci (45.3%) harbored 62 established AAM IVs and 64 eBMD IVs, while 40 ANM-eBMD loci harbored 27 AAM IVs and 25 eBMD IVs for subsequent MR analyses. Annotation of the pleiotropic SNPs by VEP and 3DSNP yielded a total of 355 and 160 genes for AAM-eBMD and ANM-eBMD, respectively. The annotation details are shown in Supplementary Tables S7, S8, S9, and S10.

**Table 1:** Novel pleiotropic loci between menstrual traits and eBMD identified by CPASSOC.

SNP	CHR	BP	A1	A2	Beta		*P*_trait_	*P*_eBMD_	*P*_CPASSOC_	PPH4	Linear closest gene*	3D interacting^†^
					Trait	eBMD						
**AAM and eBMD**												
rs115024193	4	79,714,654	A	T	0.040	0.020	7.43 × 10^−6^	4.70 × 10^−6^	1.07 × 10^−9^	0.34	*BMP2K*	*BMP2K, LOC100505702*
rs7097461	10	67,255,662	A	G	−0.014	0.008	1.90 × 10^−6^	4.80 × 10^−6^	2.49 × 10^−10^	0.78	*LRRTM3*	*-*
rs6506519	18	7,607,126	A	G	−0.024	0.014	5.27 × 10^−7^	1.30 × 10^−6^	2.05 × 10^−11^	0.91	*PTPRM*	*PTPRM*
**ANM and eBMD**												
rs4714481	6	41,521,110	T	C	0.065	0.010	1.76 × 10^−6^	7.80 × 10^−7^	5.92 × 10^−10^	0.92	*FOXP4, FOXP4-AS1*	*MIR4641, FOXP4 and MDFI*
rs147616502	16	15,686,994	T	G	−.066	0.010	4.54 × 10^−6^	5.60 × 10^−6^	9.50 × 10^−9^	0.34	*C16orf45, KIAA0430*	*KIAA0430, C16orf45 and MIR484*

SNP, single-nucleotide polymorphism; CHR, chromosome; BP, physical position of SNP, build GRCh37; A1, effect allele; A2, other allele; PPH4, posterior probability of H4 (the shared loci colocalized at the same causal variant) obtained from colocalization analysis; AAM, age at menarche; ANM, age at natural menopause; eBMD, heel estimated bone mineral density.

* Linear closest genes of index SNPs were mapped by using VEP.

^†^ 3D interacting genes of index SNPs were mapped by using 3DSNP.

By conducting colocalization analysis, we determined 22.7% (46/203) of AAM-eBMD shared loci and 27.3% (21/77) of ANM-eBMD shared loci to colocalize at the same candidate SNPs (PPH4 >0.8) (Supplementary Tables S11 and S12). Notably, one novel AAM-eBMD shared SNP (rs6506519) and one novel ANM-eBMD shared SNP (rs4714481) were colocalized, implying the same underlying causal variants influence both reproductive timing and bone health. SNP rs4714481, with the highest PPH4, is located near *FOXP4* and *FOXP4-AS1*. The former gene belongs to the forkhead box subgroup P (FOXP) of transcription factors and is associated with neurogenesis and carcinogenesis (40), while the latter is a long non-coding RNA regulating the progression of various cancers (41, 42, 43). SNP rs6506519 was mapped to *PTPRM*, whose high expression has been found to promote cervical cancer cell proliferation, migration, invasion, and lymphangiogenesis (44), while potentially acting as a tumor suppressor in epithelial ovarian cancer (45).

### Fine mapping credible set analysis

Fine mapping analysis determined a 99% credible set of causal variants for each pleiotropic locus detected by CPASSOC, facilitating downstream experiments with more potential targets. We found a total of 6,571 candidate causal SNPs for all loci shared by AAM and eBMD, and 2,980 candidate causal SNPs for all loci shared by ANM and eBMD (Supplementary Tables S13 and S14). In particular, there were 23 credible sets for AAM-eBMD and 12 credible sets for ANM-eBMD including only one SNP, indicating that these variants are highly likely to be the actual genetic causes rather than indirect markers.

### Gene-based analysis

We first identified an independent set of associated genes for each trait through MAGMA gene-based analysis, including 1,456 genes for AAM, 743 genes for ANM, and 2,204 genes for eBMD (Supplementary Tables S15, S16, S17). By intersecting AAM-associated and ANM-associated genes with the eBMD-associated genes, we found 409 overlapping genes between AAM and eBMD, as well as 179 overlapping genes between ANM and eBMD (Supplementary Table S18).

The overlap was more pronounced between MAGMA-identified genes and IVs for MR analyses, with 397 AAM-eBMD shared genes (97.1%) located within 500 kb of 186 established AAM IVs and 240 eBMD IVs. For ANM-eBMD shared genes, 169 (94.9%) of them were proximate to 53 ANM IVs and 105 eBMD IVs (Supplementary Table S18).

### Pathway and tissue-specific enrichment analysis

Pleiotropic gene sets were derived from CPASSOC and MAGMA results. The AAM-eBMD set included 641 unique genes (355 CPASSOC, 409 MAGMA, 123 overlapping), while the ANM-eBMD set contained 294 unique genes (160 CPASSOC, 178 MAGMA, 44 overlapping). These gene sets were then subjected to pathway and tissue-specific enrichment analyses.

Pleiotropic genes for AAM-eBMD demonstrated tissue-specific enrichment across brain-related tissues, heart, cervix, kidney cortex, liver, skeletal muscle tissues, skin, thyroid, ovary, and uterus (Supplementary Fig. S2). GO biological process analysis revealed significant enrichment in transcriptional regulatory processes (e.g., positive regulation of RNA metabolic process and negative regulation of RNA biosynthetic process). KEGG pathway analysis identified significant enrichment in Cushing syndrome and endocytosis pathways, while cortisol synthesis and GnRH secretion pathways exhibited high enrichment ratios but failed to reach significance after multiple testing correction (Supplementary Table S19).

For ANM-eBMD pleiotropic genes, tissue enrichment results were largely consistent with those for AAM-eBMD shared genes (Supplementary Fig. S2). The GO analysis found bio-processes related to the regulation of virus symbiosis (e.g., regulation of viral release from host cell, viral release from host cell, and exit from host). The KEGG analysis indicated significant enrichment in immune-related pathways (i.e., antigen processing and presentation, allograft rejection, graft-versus-host disease, type I diabetes mellitus, autoimmune thyroid disease, viral myocarditis) and cellular senescence (Supplementary Table S20).

### Mendelian randomization analysis

**Figure 5:**
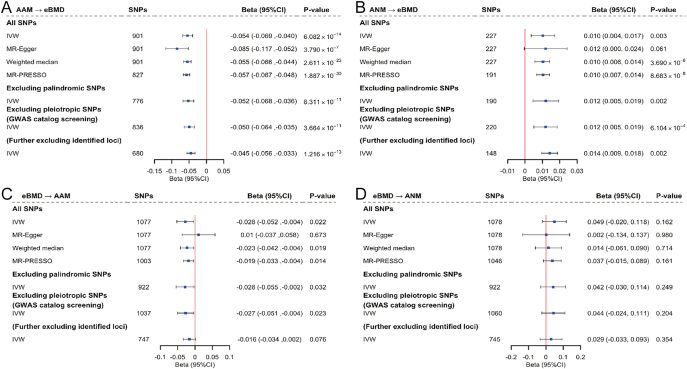
Bidirectional Mendelian randomization analysis between menstrual traits and eBMD. Panel A (AAM on eBMD) and Panel B (ANM on eBMD) show the estimates of causal associations, while Panel C (eBMD on AAM) and Panel D (eBMD on ANM) show the estimates of reverse-direction causal associations. Boxes represent the point estimates of the causal effect, and error bars represent 95% confidence intervals. IVW was applied as the primary analysis. MR-Egger, weighted median, MR-PRESSO, excluding palindromic SNPs, and excluding pleiotropic SNPs (from GWAS catalog screening or our identification results) were adopted as sensitivity analyses. AAM, age at menarche; ANM, age at natural menopause; eBMD, heel estimated bone mineral density; CI, confidence interval; IVW, inverse variance weighted.

We utilized 901 SNPs for AAM and 227 SNPs for ANM as IVs in the primary MR analyses. We first assessed the strength of IVs and confirmed that *F*-statistics for all IVs were larger than 10, indicating strong instruments (Supplementary Tables S21 and S22). Using IVW, we found that each year increase in AAM was significantly associated with a 0.054 SD decrease in eBMD (*β* = −0.054, 95% CI = −0.069 to −0.040, *P* = 6.08 × 10^−14^), and each year increase in ANM was significantly associated with a 0.01-SD increase in eBMD (*β* = 0.010, 95% CI = 0.004–0.017, *P* = 0.003). The estimates derived from IVW were consistent with MR-Egger regression, weighted median, and MR-PRESSO, as well as the sensitivity analyses excluding pleiotropic or palindromic SNPs (Fig. 5A and B). Sensitivity analyses utilizing GWAS summary statistics from non-overlapping samples corroborated our primary analysis (Table S23), indicating that sample overlap introduced minimal bias to our results. Leave-one-out analysis demonstrated the absence of outlying variants (Supplementary Fig. S3). MVMR indicated the causal relationships between menstrual traits and eBMD to be independent of common confounders (Supplementary Fig. S4).

1,077 and 1,078 SNPs were used as IVs for eBMD when the outcome was AAM and ANM, respectively (Supplementary Tables S24 and S25). Although reverse-direction MR analysis detected a modest effect of genetically predicted eBMD on AAM, this association seemed to be driven by horizontal pleiotropy (*β*_MR-Egger_ = 0.01, 95% CI = −0.037–0.058, *P*_MR-Egger_ = 0.673) and became non-significant after excluding all pleiotropic SNPs (Fig. 5C). No evidence of reverse causality was found for genetically predicted eBMD and ANM (Fig. 5D).

### Supplementary analysis

We employed female-specific eBMD GWAS data to replicate the initial findings by re-running the analytical pipeline. We observed significant genetic correlations that were consistent with those derived from sex-combined analyses (AAM *r*_g_ = −0.068, *P* = 2.62 × 10^−5^; ANM *r*_g_ = 0.060, *P* = 0.022). All ten genomic regions with significant AAM-eBMD correlations in the primary analysis demonstrated directionally concordant associations and maintained statistical significance (*P* < 0.05). Similarly, 10 of 12 ANM-eBMD local genetic correlations showed consistent directional effects (*P* < 0.05) (Supplementary Tables S2 and S3). For pleiotropic loci detected by CPASSOC, the female-specific analysis confirmed all 203 AAM-eBMD signals at *P* < 0.05, with 146 signals (71.9%) maintaining genome-wide significance (*P* < 5 × 10^−8^). For ANM-eBMD shared loci, 38 of 77 signals (49.4%) were replicated (*P* < 0.05), and 24 achieved genome-wide significance (Supplementary Tables S4 and S5). In gene-based analyses by MAGMA, 2,148 of 2,204 eBMD-associated genes (97.5%) exhibited directionally consistent associations in the female-specific analysis, with 1,700 genes achieving statistical significance (*P* < 0.05), including 312 AAM-eBMD and 147 ANM-eBMD shared genes (Supplementary Table S17). Female-specific MR analysis also showed a consistent negative effect of genetically predicted AAM on eBMD (*β* = −0.046, 95% CI = −0.065 to −0.027, *P* = 1.63 × 10^−6^) and a positive effect of genetically predicted ANM on eBMD (*β* = 0.024, 95% CI = 0.016–0.032, *P* = 1.62 × 10^−9^).

## Discussion

To the best of our knowledge, this is the first large-scale genome-wide cross-trait analysis that comprehensively investigates the shared architecture between menstrual traits and eBMD. We identified intrinsic links underlying AAM, ANM, and eBMD, as demonstrated by both globally and locally shared heritability. We further decomposed such genetic overlaps into pleiotropy and causality, reflected by shared loci and genes identified in CPASSOC or MAGMA, and the putative causal relationship demonstrated by MR.

With improved power from GWAS datasets of enlarged sample size, we found modest but significant genetic correlations between the two menstrual traits and eBMD, largely consistent in direction and magnitude with results from previous LDSC analyses (8, 14). The moderate SNP-based heritability for these traits might inherently constrain the magnitude of genetic correlations that could be detected. The global genetic correlations were further supported by the identification of significant local signals across the genome, which collectively underscore the substantial shared biological foundation. Estimates of regional *r*_g_ were largely in line with our global *r*_g_ and MR analyses, given that eight out of the ten identified AAM-eBMD shared regions presented negative associations, and half of the ANM-eBMD shared regions presented positive associations.

Both pleiotropy and causality can contribute to the significant overall genetic overlap, and we confirmed these two possibilities in downstream analyses. Through cross-trait meta-analysis, we identified 203 AAM-eBMD shared loci and 77 ANM-eBMD shared loci, among which 5 were novel and might shed light on common etiology. We found a moderate proportion of loci showed strong evidence of colocalization (PPH4 >0.8, two out of five). The locus with the highest PPH4 (indexed by SNP rs4714481) was shared by ANM and eBMD and located near *FOXP4* and *FOXP4-AS1*. The carcinogenesis role of these two genes has been extensively studied, while their relationship with ANM or eBMD remains elusive. The colocalized locus indexed by SNP rs6506519 was shared between AAM and eBMD. This SNP maps to *PTPRM*, which exhibits tissue-specific roles in cancer, promoting tumorigenesis in cervical cancer but suppressing it in colorectal, neuroendocrine, and ovarian cancers. However, *PTPRM*’s regulatory mechanisms in AAM and eBMD remain unexplored. In addition, SNP rs7097461 near *LRRTM3* approached colocalization criteria (PPH4 = 0.78). Notably, both *LRRTM3* and *PTPRM* showed high HuGE scores for BMI (indicating strong genetic evidence for these genes’ involvement in BMI regulation) (46), which positively correlates with BMD (47, 48), suggesting these genes may influence bone metabolism through BMI-mediated pathways. Moreover, increased childhood BMI is associated with earlier AAM (49), complicating the interplay between AAM and eBMD.

With a reduced burden of multiple testing and enhanced statistical power, our gene-based analyses identified more pleiotropic loci (409 for AAM-eBMD and 179 for ANM-eBMD) than CPASSOC. An interesting example is *WNT10B*, a pleiotropic gene shared between AAM and eBMD, whose roles in the normal development of multiple tissues have been well documented (50). Although SNPs of *WNT10B* have been found to influence BMD through candidate-gene-based approaches (51, 52), large-scale GWAS failed to detect any correlation between *WNT10B* and bone properties (50), likely due to the modest effects of individual SNPs. Among pleiotropic genes shared between ANM and eBMD, a notable portion (*NBEAL2*, *SETD2*, *AIF1*, *HSPA1A*, *HSPA1B*, *LSM2*, *NELFE*, *LST1*, *NKAPL*, *EBPL*, *IFI35*, and *PTGES3L*) were implicated in autoimmune inflammatory responses and diseases (53, 54, 55, 56, 57, 58, 59, 60, 61), highlighting an important role of autoimmune processes in the pathophysiology of osteoporosis. The initiation and perpetuation of autoimmunity are recognized to be triggered by innate immunity (62). Intriguingly, three members (*TRIM8*, *TRIM26*, and *TRIM31*) of the tripartite motif (TRIM) family, known regulators of innate immunity (63), were also pleiotropic genes shared by ANM and eBMD. Our findings, together with recent studies showing the involvement of part of the TRIM family members in osteoblast differentiation and bone remodeling (64, 65), underscore the importance of a thorough assessment of this family’s roles in osteoporosis.

We adopted two complementary methods, CPASSOC and MAGMA, to identify pleiotropic loci and genes between menstrual traits and eBMD. For AAM-eBMD, we identified a total of 641 genes, of which 123 (19.2%) were detected by both methods. For ANM-eBMD, 294 genes were identified, with 44 genes (15.0%) showing overlap between the two approaches. The modest overlap (15–19%) suggests that pleiotropy between menstrual traits and eBMD operates through diverse mechanisms–some driven by individual variants with large pleiotropic effects (captured by CPASSOC), others by polygenic architectures where multiple variants within the same gene contribute to pleiotropy (captured by MAGMA). This methodological complementarity strengthens our findings by providing a comprehensive view of the pleiotropic landscape.

We further explored potential biological pathways in which those shared genes participated through pathway enrichment analyses. Regarding AAM-eBMD shared genes, we found significant enrichment in GO terms of transcriptional regulatory processes. Previous studies have demonstrated that estrogen stimulates rapid bone mineral deposition through transcriptional regulation, with estrogen receptors functioning as transcription factors that control GnRH production and bone morphogenetic protein expression during the critical post-menarche period (66, 67). Consistently, KEGG analysis revealed additional enrichment in hormonal regulatory pathways, suggesting these pleiotropic genes may influence both AAM and eBMD by modulating hormone-responsive gene expression during bone metabolism and reproductive maturation. Regarding ANM-eBMD shared genes, we found significant enrichment in immune-related diseases and cellular senescence, underlining the role of immune mechanisms in bone changes. It has been recognized that, in addition to the direct negative effect of estrogen deficiency on bone, the indirect effect of altered immune status in postmenopausal women also contributes to bone destruction (68). For instance, estrogen deficiency and increased senescent cells in postmenopausal women both lead to elevated levels of pro-inflammatory cytokines and immune cells, responsible for bone loss (68, 69, 70).

Our comprehensive two-sample MR demonstrated that genetically predicted later AAM or earlier ANM was causally linked to a decreased level of eBMD. While largely concordant with results of existing MR studies, our study extended previous findings with improved accuracy by utilizing i) the latest and largest GWAS of menstrual traits and eBMD, ii) an expanded set of complementary analyses to validate the MR model assumptions, and iii) an extensive MVMR design to assess the independent effect of AAM or ANM. The estimated causal effects remained directionally consistent across a variety of sensitivity analyses, as well as after adjustment for multiple confounders, reinforcing the robustness of our MR findings. Later AAM and earlier ANM are correlated with a shorter duration of estrogen exposure, and thus our MR results indirectly suggest a potential causal effect of estrogen exposure on BMD. The GWAS of more accurate lifelong estrogen exposure, accounting for pregnancy and hormone use, would help further validate this causal relationship.

Our study provides genome-wide insights into the shared genetic landscape between menstrual timing and eBMD, with significant translational potential. First, our findings enhance early screening and risk stratification for osteoporosis. The identification of pleiotropic genetic variants associated with both menstrual traits and eBMD paves the way for the development of more precise and targeted risk assessment tools (such as polygenic risk scores), tailored specifically for women. These genetic markers can also be integrated into existing prediction models that rely on conventional risk factors, enabling more accurate and individualized risk stratification. Second, our results hold promise for guiding clinical practice by enabling anticipatory interventions to prevent osteoporosis. For instance, women with a history of late menarche or indicators of early menopause could benefit from more proactive bone health monitoring and earlier lifestyle or therapeutic interventions to reduce the risk of future bone loss. Finally, the shared genetic variants and biological pathways uncovered in our study present compelling opportunities for novel therapeutic strategies. Genes, small molecules, and bioprocesses affecting both menstrual traits and BMD may play pivotal roles in the etiology of osteoporosis. Targeting these shared pathways could lead to more effective treatments for osteoporosis and hopefully address related reproductive disorders at the same time.

Several limitations need to be acknowledged. First, our main results were generated using sex-combined eBMD GWAS. However, supplementary analyses were undertaken to assess the potential effect of sex heterogeneity and showed minimal influence. Future sufficiently powered female-specific data are warranted to provide unbiased results. Second, genetic data for both menstrual traits and eBMD involved UKB participants (sample overlap ranging from 17 to 53% across all trait pairs), which may exacerbate bias due to weak instruments and winner’s curse in MR results. However, we ensured that all IVs used in MR analyses were not weak instruments (*F*-statistics >10). Furthermore, our sensitivity analysis using the summary statistics of IVs from non-overlapping samples yielded consistent results. Overall, these measures strengthen the credibility of our MR results and mitigate potential biases arising from sample overlap. Third, genetic data analyzed in this study were exclusively from European ancestry, limiting the generalizability of results to other ethnic populations. Finally, the genes, pathways, and tissues identified by our study relied entirely on functional datasets and algorithms; in-depth experimental studies are warranted to reveal underlying mechanisms.

## Conclusion

To summarize, our study extends previously reported associations between menstrual traits and osteoporosis by providing evidence of genetic correlations, pleiotropic loci, and potential causal effects of menstrual traits on eBMD. Our findings emphasize the pleiotropy and potential biological mechanisms linking these traits and could be instrumental for predicting osteoporosis risk and formulating novel pharmaceutical interventions.

## Supplementary materials





## Declaration of interest

The authors declare that there is no conflict of interest that could be perceived as prejudicing the impartiality of the work reported.

## Funding

This study was supported by the Science Fund for Creative Research Groups of Science and Technology Bureau of Sichuan Province (2024NSFTD0030), the Recruitment Program for Young Professionals of China, the Promotion Plan for Basic Medical Sciences and the Development Plan for Cutting-Edge Disciplines, Sichuan University. The funders of this study had no role in study design, data collection, data analysis, data interpretation, writing of the report, or the decision for submission.

## Author contribution statement

XJ, BL, LH, and YJ conceived and designed the study. BL, XS, BY, LQ, and MT contributed to the analysis of the data. BL, YQ, XW and XJ drafted the manuscript. LZ, YZ, LW, JX, YC, SW, and SC contributed to the interpretations of the findings and the critical revision of the article. WW, LS, MF, JL, BZ, YJ, and XJ supervised the study. XJ is the guarantor of this manuscript.

## Data availability

GWAS summary statistics for heel bone mineral density, age at menarche, and age at menopause are publicly available from GEFOS (http://www.gefos.org/?q=content/data-release-2018), Cambridge Repository (https://doi.org/10.17863/CAM.107943), and ReproGen (https://reprogen.org/data_download.html), respectively.

## Ethics approval and consent to participate

This study performed secondary analyses using publicly available GWAS summary statistics, which are at the aggregated level without personal identifiers. The original GWAS studies obtained ethical approval from their respective ethics review boards and informed consent from participants.

## References

[1] Chang HK, Chang DG, Myong JP, et al. Bone mineral density among Korean females aged 20–50 years: influence of age at menarche (The Korea National Health and Nutrition Examination Survey 2008–2011). Osteoporos Int 2017 28 2129–2136. (10.1007/s00198-017-3997-0) 28293690

[2] Yang Y, Wang S & Cong H. Association between age at menarche and bone mineral density in postmenopausal women. J Orthop Surg Res 2023 18 51. (10.1186/s13018-023-03520-2) 36650576 PMC9843934

[3] Kuh D, Muthuri S, Cooper R, et al. Menopause, reproductive life, hormone replacement therapy, and bone phenotype at age 60–64 years: a British birth cohort. J Clin Endocrinol Metab 2016 101 3827–3837. (10.1210/jc.2016-1828) 27472291 PMC5052353

[4] Shieh A, Ruppert KM, Greendale GA, et al. Associations of age at menopause with postmenopausal bone mineral density and fracture risk in women. J Clin Endocrinol Metab 2022 107 e561–e569. (10.1210/clinem/dgab690) 34537850 PMC8764341

[5] Zhu Z, Hasegawa K, Camargo CA, et al. Investigating asthma heterogeneity through shared and distinct genetics: insights from genome-wide cross-trait analysis. J Allergy Clin Immunol 2021 147 796–807. (10.1016/j.jaci.2020.07.004) 32693092 PMC7368660

[6] Farahmand M, Rahmati M, Azizi F, et al. The impact of endogenous estrogen exposure duration on fracture incidence: a longitudinal cohort study. J Clin Endocrinol Metab 2022 107 e3321–e3329. (10.1210/clinem/dgac248) 35512228

[7] Bulik-Sullivan B, Finucane HK, Anttila V, et al. An atlas of genetic correlations across human diseases and traits. Nat Genet 2015 47 1236–1241. (10.1038/ng.3406) 26414676 PMC4797329

[8] Trajanoska K, Morris JA, Oei L, et al. Assessment of the genetic and clinical determinants of fracture risk: genome wide association and mendelian randomisation study. BMJ 2018 362 k3225. (10.1136/bmj.k3225) 30158200 PMC6113773

[9] Zhang Q, Greenbaum J, Zhang WD, et al. Age at menarche and osteoporosis: a Mendelian randomization study. Bone 2018 117 91–97. (10.1016/j.bone.2018.09.015) 30240960 PMC6346741

[10] Cousminer DL, Mitchell JA, Chesi A, et al. Genetically determined later puberty impacts lowered bone mineral density in childhood and adulthood. J Bone Miner Res 2018 33 430–436. (10.1002/jbmr.3320) 29068475 PMC5839967

[11] Ruth KS, Day FR, Hussain J, et al. Genetic insights into biological mechanisms governing human ovarian ageing. Nature 2021 596 393–397. (10.1038/s41586-021-03779-7) 34349265 PMC7611832

[12] Day FR, Thompson DJ, Helgason H, et al. Genomic analyses identify hundreds of variants associated with age at menarche and support a role for puberty timing in cancer risk. Nat Genet 2017 49 834–841. (10.1038/ng.3841) 28436984 PMC5841952

[13] Morris JA, Kemp JP, Youlten SE, et al. An atlas of genetic influences on osteoporosis in humans and mice. Nat Genet 2019 51 258–266. (10.1038/s41588-018-0302-x) 30598549 PMC6358485

[14] Day FR, Ruth KS, Thompson DJ, et al. Large-scale genomic analyses link reproductive aging to hypothalamic signaling, breast cancer susceptibility and BRCA1-mediated DNA repair. Nat Genet 2015 47 1294–1303. (10.1038/ng.3412) 26414677 PMC4661791

[15] Hendrickx G, Boudin E & Van Hul W. A look behind the scenes: the risk and pathogenesis of primary osteoporosis. Nat Rev Rheumatol 2015 11 462–474. (10.1038/nrrheum.2015.48) 25900210

[16] Burgess S & Thompson SG. Multivariable Mendelian randomization: the use of pleiotropic genetic variants to estimate causal effects. Am J Epidemiol 2015 181 251–260. (10.1093/aje/kwu283) 25632051 PMC4325677

[17] Kentistou KA, Kaisinger LR, Stankovic S, et al. Understanding the genetic complexity of puberty timing across the allele frequency spectrum. Nat Genet 2024 56 1397–1411. (10.1038/s41588-024-01798-4) 38951643 PMC11250262

[18] Zhang Y, Lu Q, Ye Y, et al. SUPERGNOVA: local genetic correlation analysis reveals heterogeneous etiologic sharing of complex traits. Genome Biol 2021 22 262. (10.1186/s13059-021-02478-w) 34493297 PMC8422619

[19] Zhu X, Feng T, Tayo BO, et al. Meta-analysis of correlated traits via summary statistics from GWASs with an application in hypertension. Am J Hum Genet 2015 96 21–36. (10.1016/j.ajhg.2014.11.011) 25500260 PMC4289691

[20] Purcell S, Neale B, Todd-Brown K, et al. PLINK: a tool set for whole-genome association and population-based linkage analyses. Am J Hum Genet 2007 81 559–575. (10.1086/519795) 17701901 PMC1950838

[21] Martin FJ, Amode MR, Aneja A, et al. Ensembl 2023. Nucleic Acids Res 2023 51 D933–D941. (10.1093/nar/gkac958) 36318249 PMC9825606

[22] Quan C, Ping J, Lu H, et al. 3DSNP 2.0: update and expansion of the noncoding genomic variant annotation database. Nucleic Acids Res 2022 50 D950–D955. (10.1093/nar/gkab1008) 34723317 PMC8728236

[23] Giambartolomei C, Vukcevic D, Schadt EE, et al. Bayesian test for colocalisation between pairs of genetic association studies using summary statistics. PLoS Genet 2014 10 e1004383. (10.1371/journal.pgen.1004383) 24830394 PMC4022491

[24] Farh KKH, Marson A, Zhu J, et al. Genetic and epigenetic fine mapping of causal autoimmune disease variants. Nature 2015 518 337–343. (10.1038/nature13835) 25363779 PMC4336207

[25] de Leeuw CA, Mooij JM, Heskes T, et al. MAGMA: generalized gene-set analysis of GWAS data. PLoS Comput Biol 2015 11 e1004219. (10.1371/journal.pcbi.1004219) 25885710 PMC4401657

[26] Liao Y, Wang J, Jaehnig EJ, et al. WebGestalt 2019: gene set analysis toolkit with revamped UIs and APIs. Nucleic Acids Res 2019 47 W199–W205. (10.1093/nar/gkz401) 31114916 PMC6602449

[27] Watanabe K, Taskesen E, van Bochoven A, et al. Functional mapping and annotation of genetic associations with FUMA. Nat Commun 2017 8 1826. (10.1038/s41467-017-01261-5) 29184056 PMC5705698

[28] Burgess S, Scott RA, Timpson NJ, et al. Using published data in Mendelian randomization: a blueprint for efficient identification of causal risk factors. Eur J Epidemiol 2015 30 543–552. (10.1007/s10654-015-0011-z) 25773750 PMC4516908

[29] Bowden J, Davey Smith G, Haycock PC, et al. Consistent estimation in Mendelian randomization with some invalid instruments using a weighted median estimator. Genet Epidemiol 2016 40 304–314. (10.1002/gepi.21965) 27061298 PMC4849733

[30] Bowden J, Davey Smith G & Burgess S. Mendelian randomization with invalid instruments: effect estimation and bias detection through Egger regression. Int J Epidemiol 2015 44 512–525. (10.1093/ije/dyv080) 26050253 PMC4469799

[31] Verbanck M, Chen CY, Neale B, et al. Detection of widespread horizontal pleiotropy in causal relationships inferred from Mendelian randomization between complex traits and diseases. Nat Genet 2018 50 693–698. (10.1038/s41588-018-0099-7) 29686387 PMC6083837

[32] Pulit SL, Stoneman C, Morris AP, et al. Meta-analysis of genome-wide association studies for body fat distribution in 694 649 individuals of European ancestry. Hum Mol Genet 2019 28 166–174. (10.1093/hmg/ddy327) 30239722 PMC6298238

[33] Wootton RE, Richmond RC, Stuijfzand BG, et al. Evidence for causal effects of lifetime smoking on risk for depression and schizophrenia: a Mendelian randomisation study. Psychol Med 2020 50 2435–2443. (10.1017/S0033291719002678) 31689377 PMC7610182

[34] Klimentidis YC, Raichlen DA, Bea J, et al. Genome-wide association study of habitual physical activity in over 377,000 UK Biobank participants identifies multiple variants including CADM2 and APOE. Int J Obes 2018 42 1161–1176. (10.1038/s41366-018-0120-3) PMC6195860 29899525

[35] Lee JJ, Wedow R, Okbay A, et al. Gene discovery and polygenic prediction from a genome-wide association study of educational attainment in 1.1 million individuals. Nat Genet 2018 50 1112–1121. (10.1038/s41588-018-0147-3) 30038396 PMC6393768

[36] Elks CE, Perry JRB, Sulem P, et al. Thirty new loci for age at menarche identified by a meta-analysis of genome-wide association studies. Nat Genet 2010 42 1077–1085. (10.1038/ng.714) 21102462 PMC3140055

[37] Perry JR, Day F, Elks CE, et al. Parent-of-origin-specific allelic associations among 106 genomic loci for age at menarche. Nature 2014 514 92–97. (10.1038/nature13545) 25231870 PMC4185210

[38] Zhang L, Wei XT, Niu JJ, et al. Joint genome-wide association analyses identified 49 novel loci for age at natural menopause. J Clin Endocrinol Metab 2021 106 2574–2591. (10.1210/clinem/dgab377) 34050765

[39] Kichaev G, Bhatia G, Loh PR, et al. Leveraging polygenic functional enrichment to improve GWAS power. Am J Hum Genet 2019 104 65–75. (10.1016/j.ajhg.2018.11.008) 30595370 PMC6323418

[40] Gao C, Zhu H, Gong P, et al. The functions of FOXP transcription factors and their regulation by post-translational modifications. Biochim Biophys Acta Gene Regul Mech 2023 1866 194992. (10.1016/j.bbagrm.2023.194992) 37797785

[41] Hua T, Tian YJ, Wang RM, et al. FOXP4-AS1 is a favorable prognostic-related enhancer RNA in ovarian cancer. Biosci Rep 2021 41 BSR20204008. (10.1042/BSR20204008) 33870423 PMC8150160

[42] Xiong J, Wu L, Huang L, et al. LncRNA FOXP4-AS1 promotes progression of ewing sarcoma and is associated with immune infiltrates. Front Oncol 2021 11 718876. (10.3389/fonc.2021.718876) 34765540 PMC8577041

[43] Luo X, Gao Q, Zhou T, et al. FOXP4-AS1 inhibits papillary thyroid carcinoma proliferation and migration through the AKT signaling pathway. Front Oncol 2022 12 900836. (10.3389/fonc.2022.900836) 35720005 PMC9202991

[44] Liu P, Zhang C, Liao Y, et al. High expression of PTPRM predicts poor prognosis and promotes tumor growth and lymph node metastasis in cervical cancer. Cell Death Dis 2020 11 687. (10.1038/s41419-020-02826-x) 32826853 PMC7443137

[45] Li X, Ding W, Rao Y, et al. Role of protein tyrosine phosphatase receptor type M in epithelial ovarian cancer progression. J Ovarian Res 2023 16 131. (10.1186/s13048-023-01220-3) 37403117 PMC10318840

[46] Westendorf JJ, Bonewald LF, Kiel DP, et al. The musculoskeletal knowledge portal: improving access to multi-omics data. Nat Rev Rheumatol 2022 18 1–2. (10.1038/s41584-021-00711-1) 34686856 PMC9346538

[47] Rodrick E & Kindler JM. Bone mass accrual in children. Curr Opin Endocrinol Diabetes Obes 2024 31 53–59. (10.1097/MED.0000000000000849) 38010050 PMC11015822

[48] Larsson SC & Burgess S. Causal role of high body mass index in multiple chronic diseases: a systematic review and meta-analysis of Mendelian randomization studies. BMC Med 2021 19 320. (10.1186/s12916-021-02188-x) 34906131 PMC8672504

[49] Wang L, Xu F, Zhang Q, et al. Causal relationships between birth weight, childhood obesity and age at menarche: a two-sample Mendelian randomization analysis. Clin Endocrinol 2023 98 212–220. (10.1111/cen.14831) 36237121

[50] Perkins RS, Singh R, Abell AN, et al. The role of WNT10B in physiology and disease: a 10-year update. Front Cell Dev Biol 2023 11 1120365. (10.3389/fcell.2023.1120365) 36814601 PMC9939717

[51] Zmuda JM, Yerges LM, Kammerer CM, et al. Association analysis of WNT10B with bone mass and structure among individuals of African ancestry. J Bone Miner Res 2009 24 437–447. (10.1359/jbmr.081106) 19016593 PMC2659518

[52] Van Camp JK, Beckers S, Zegers D, et al. Genetic association study of WNT10B polymorphisms with BMD and adiposity parameters in Danish and Belgian males. Endocrine 2013 44 247–254. (10.1007/s12020-012-9869-7) 23325361

[53] Delage L, Carbone F, Riller Q, et al. NBEAL2 deficiency in humans leads to low CTLA-4 expression in activated conventional T cells. Nat Commun 2023 14 3728. (10.1038/s41467-023-39295-7) 37349339 PMC10287742

[54] Chen Y, Chen K, Zhu H, et al. Methyltransferase Setd2 prevents T cell-mediated autoimmune diseases via phospholipid remodeling. Proc Natl Acad Sci U S A 2024 121 e2314561121. (10.1073/pnas.2314561121) 38359295 PMC10895270

[55] De Leon-Oliva D, Garcia-Montero C, Fraile-Martinez O, et al. AIF1: function and connection with inflammatory diseases. Biology 2023 12 694. (10.3390/biology12050694) 37237507 PMC10215110

[56] Parkes JE, Rothwell S, Day PJ, et al. Systematic protein-protein interaction and pathway analyses in the idiopathic inflammatory myopathies. Arthritis Res Ther 2016 18 156. (10.1186/s13075-016-1061-7) 27388770 PMC4936183

[57] Schott G & Garcia-Blanco MA. MHC class III RNA binding proteins and immunity. RNA Biol 2021 18 640–646. (10.1080/15476286.2020.1860388) PMC8163431 33280511

[58] Yau ACY, Tuncel J, Haag S, et al. Conserved 33-kb haplotype in the MHC class III region regulates chronic arthritis. Proc Natl Acad Sci U S A 2016 113 E3716–E3724. (10.1073/pnas.1600567113) 27303036 PMC4932949

[59] Xie G, Lu Y, Sun Y, et al. Identification of the NF-κB activating protein-like locus as a risk locus for rheumatoid arthritis. Ann Rheum Dis 2013 72 1249–1254. (10.1136/annrheumdis-2012-202076) 23223422 PMC3686260

[60] Wu C, Tan S, Liu L, et al. Transcriptome-wide association study identifies susceptibility genes for rheumatoid arthritis. Arthritis Res Ther 2021 23 38. (10.1186/s13075-021-02419-9) 33482886 PMC7821659

[61] Bianchi M, Kozyrev SV, Notarnicola A, et al. Contribution of rare genetic variation to disease susceptibility in a large Scandinavian myositis cohort. Arthritis Rheumatol 2022 74 342–352. (10.1002/art.41929) 34279065

[62] Saferding V & Blüml S. Innate immunity as the trigger of systemic autoimmune diseases. J Autoimmun 2020 110 102382. (10.1016/j.jaut.2019.102382) 31883831

[63] Kawai T & Akira S. Regulation of innate immune signalling pathways by the tripartite motif (TRIM) family proteins. EMBO Mol Med 2011 3 513–527. (10.1002/emmm.201100160) 21826793 PMC3377094

[64] Kim K, Kim JH, Kim I, et al. TRIM38 regulates NF-κB activation through TAB2 degradation in osteoclast and osteoblast differentiation. Bone 2018 113 17–28. (10.1016/j.bone.2018.05.009) 29753717

[65] Liu RX, Gu RH, Li ZP, et al. Trim21 depletion alleviates bone loss in osteoporosis via activation of YAP1/β-catenin signaling. Bone Res 2023 11 56. (10.1038/s41413-023-00296-3) 37884520 PMC10603047

[66] Otani H, Otsuka F, Takeda M, et al. Regulation of GNRH production by estrogen and bone morphogenetic proteins in GT1-7 hypothalamic cells. J Endocrinol 2009 203 87–97. (10.1677/JOE-09-0065) 19635757 PMC2768486

[67] Mills EG, Yang L, Nielsen MF, et al. The relationship between bone and reproductive hormones beyond estrogens and androgens. Endocr Rev 2021 42 691–719. (10.1210/endrev/bnab015) 33901271 PMC8599211

[68] Fischer V & Haffner-Luntzer M. Interaction between bone and immune cells: implications for postmenopausal osteoporosis. Semin Cell Dev Biol 2022 123 14–21. (10.1016/j.semcdb.2021.05.014) 34024716

[69] Föger-Samwald U, Dovjak P, Azizi-Semrad U, et al. Osteoporosis: pathophysiology and therapeutic options. EXCLI J 2020 19 1017–1037. (10.17179/excli2020-2591) 32788914 PMC7415937

[70] Okamoto K, Nakashima T, Shinohara M, et al. Osteoimmunology: the conceptual framework unifying the immune and skeletal systems. Physiol Rev 2017 97 1295–1349. (10.1152/physrev.00036.2016) 28814613

