# Supplementary material for: Genome-wide insights of shared genetic architecture between menstrual traits and bone mineral density
Source: Endocr Connect. 2025 Oct 23;14(10):e250278. doi: 10.1530/EC-25-0278 (PMC12552850; doi:10.1530/EC-25-0278)
Supplement: Supplementary file 1 [file supplementary_materials.pdf]

Supplementary Materials

Supplementary Figures 2

Figure S1. Estimated SNP-based heritability (h2) explained by the whole genome and after excluding identified pleiotropic loci. 2

Figure S2. GTEx tissue enrichment analysis of associations between menstrual traits and eBMD. 3

Figure S3. Box plot of betas in leave-one-out analysis where one SNP was removed at a time and inverse-variance weighted analysis was conducted based on the remaining SNPs. 4

Figure S4. Independent effects of genetically predicted menstrual traits on eBMD after adjusting for each potential confounder separately and together using multi-variable Mendelian randomization. 5

STROBE-MR checklist of recommended items to address in reports of Mendelian randomization studies[1 2] 6

Supplementary Figures

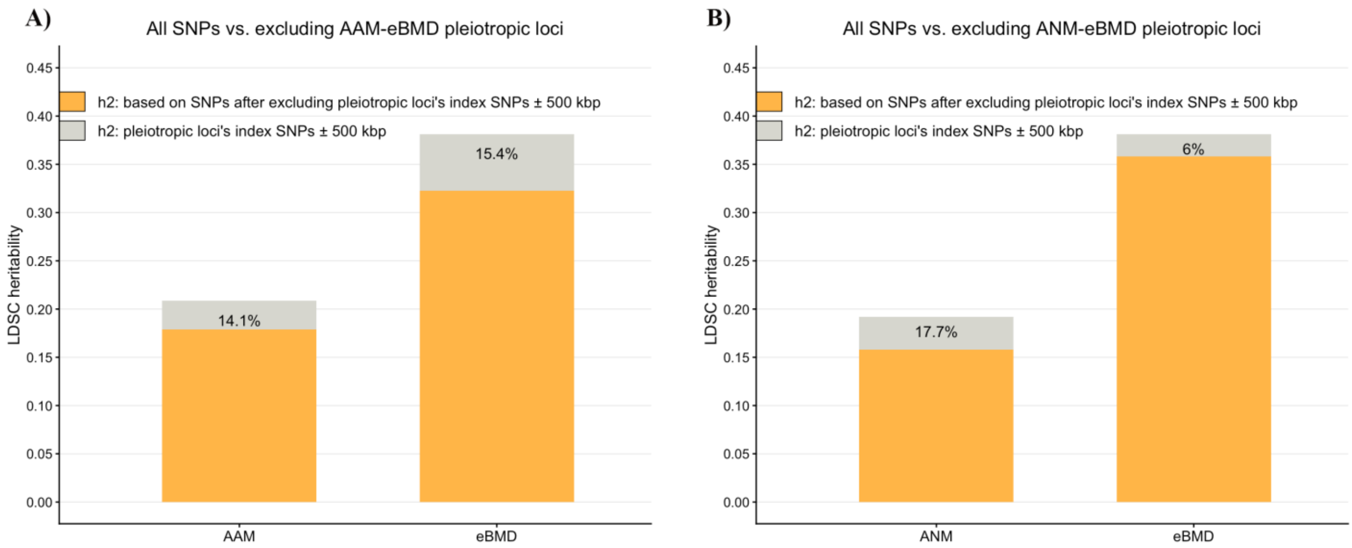

Figure S1. Estimated SNP-based heritability (h2) explained by the whole genome and after excluding identified pleiotropic loci.

The gray bar represents the proportion of SNP-based heritability (values indicated on each bar) attributable to pleiotropic loci (index SNP ±500kb surrounded areas), with the total height of the bar corresponding to the overall SNP-based heritability estimated using LDSC. (A) AAM-eBMD, (B)ANM-eBMD. AAM, age at menarche; ANM, age at menopause; eBMD, Heel-estimated bone mineral density.

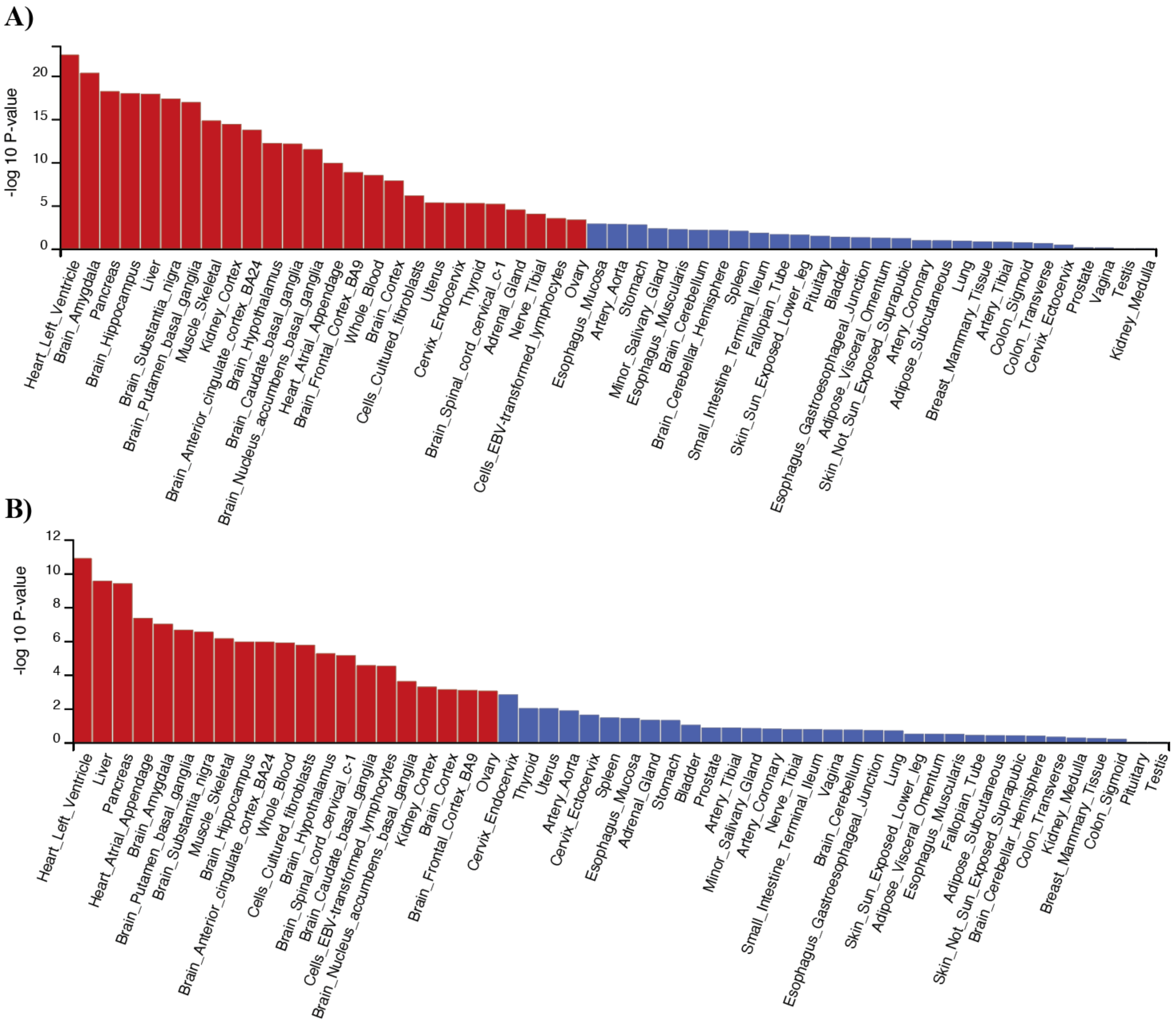

Figure S2. **GTEx tissue enrichment analysis of associations between menstrual traits and eBMD.**

The gene sets contain VEP and 3DSNP annotated CPASSOC-identified loci, and MAGMA-identified genes. (A) AAM and eBMD, (B) ANM and eBMD. AAM, age at menarche; ANM, age at natural menopause; eBMD, heel estimated bone mineral density. The red color denotes the differentially expressed genes (DEG) sets that were significantly enriched ($P_{FDR} < 0.05$), whereas the blue color represents those that were not.

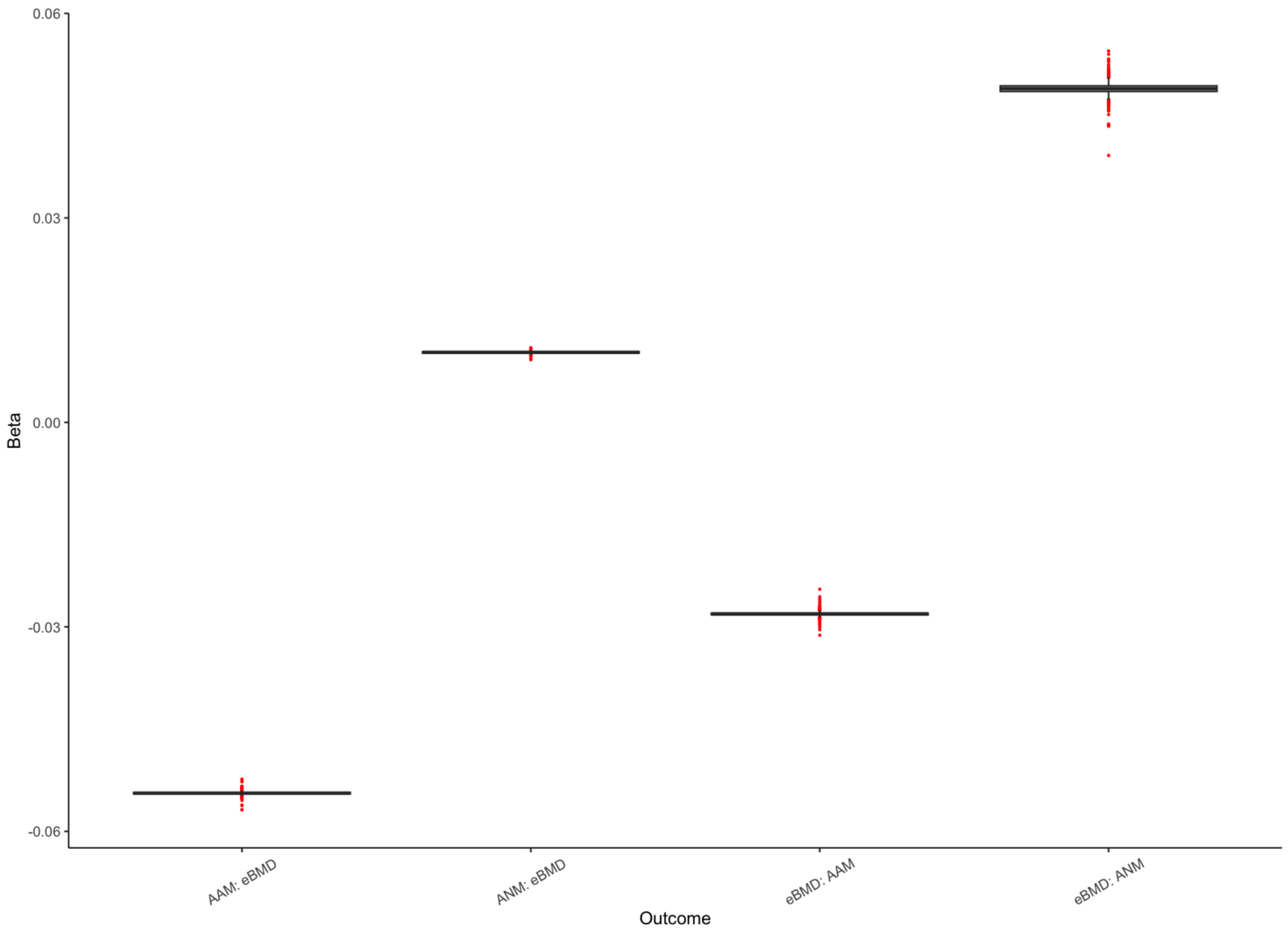

Figure S3. Box plot of betas in leave-one-out analysis where one SNP was removed at a time and inverse-variance weighted analysis was conducted based on the remaining SNPs.

Beta, effect allele beta coefficient; AAM, age at menarche; ANM, age at natural menopause; eBMD, heel estimated bone mineral density.

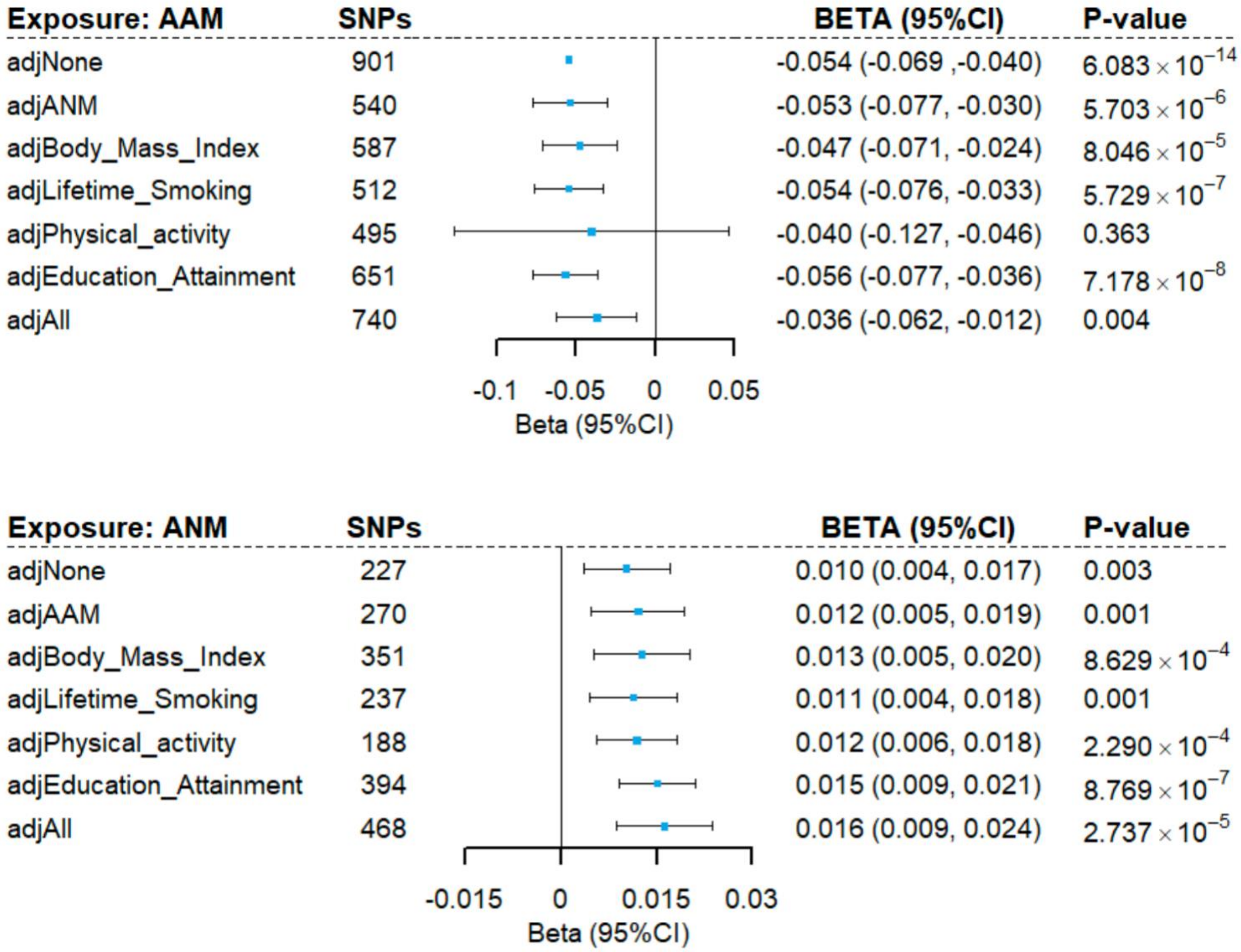

Figure S4. Independent effects of genetically predicted menstrual traits on eBMD after adjusting for each potential confounder separately and together using multi-variable Mendelian randomization.

The y-axis details the genetically predicted confounder for which adjustment was made. Boxes represent the point estimates of the causal effects of genetically predicted menstrual traits on eBMD, and error bars represent 95% confidence intervals. adjNone, the point estimates of the causal effects of genetically predicted AAM/ANM on eBMD using univariable Mendelian randomization; AAM, age at menarche; ANM, age at menopause; eBMD, heel estimated bone mineral density.

STROBE-MR checklist of recommended items to address in reports of Mendelian randomization studies[1 2]

Item No.	Section		Checklist item	Page No.
1	**TITLE and ABSTRACT**		Indicate Mendelian randomization (MR) as the study's design in the title and/or the abstract if that is a main purpose of the study	3
	**INTRODUCTION**			
2	**Background**		Explain the scientific background and rationale for the reported study. What is the exposure? Is a potential causal relationship between exposure and outcome plausible? Justify why MR is a helpful method to address the study question	5
3	**Objectives**		State specific objectives clearly, including pre-specified causal hypotheses (if any). State that MR is a method that, under specific assumptions, intends to estimate causal effects	6
	**METHODS**			
4	**Study design and data sources**		Present key elements of the study design early in the article. Consider including a table listing sources of data for all phases of the study. For each data source contributing to the analysis, describe the following:	Figure 1 and Table S1
		a)	Setting: Describe the study design and the underlying population, if possible. Describe the setting, locations, and relevant dates, including periods of recruitment, exposure, follow-up, and data collection, when available.	6 and Table S1
		b)	Participants: Give the eligibility criteria, and the sources and methods of selection of participants. Report the sample size, and whether any power or sample size calculations were carried out prior to the main analysis	6 and Table S1
		c)	Describe measurement, quality control and selection of genetic variants	6, 7
		d)	For each exposure, outcome, and other relevant variables, describe methods of assessment and diagnostic criteria for diseases	6, 7

		e)	Provide details of ethics committee approval and participant informed consent, if relevant	NA
5	**Assumptions**		Explicitly state the three core IV assumptions for the main analysis (relevance, independence and exclusion restriction) as well assumptions for any additional or sensitivity analysis	9
6	**Statistical methods: main analysis**		Describe statistical methods and statistics used	
		a)	Describe how quantitative variables were handled in the analyses (i.e., scale, units, model)	NA
		b)	Describe how genetic variants were handled in the analyses and, if applicable, how their weights were selected	6
		c)	Describe the MR estimator (e.g. two-stage least squares, Wald ratio) and related statistics. Detail the included covariates and, in case of two-sample MR, whether the same covariate set was used for adjustment in the two samples	9
		d)	Explain how missing data were addressed	NA
		e)	If applicable, indicate how multiple testing was addressed	NA
7	**Assessment of assumptions**		Describe any methods or prior knowledge used to assess the assumptions or justify their validity	9
8	**Sensitivity analyses and additional analyses**		Describe any sensitivity analyses or additional analyses performed (e.g. comparison of effect estimates from different approaches, independent replication, bias analytic techniques, validation of instruments, simulations)	9
9	**Software and pre-registration**			
		a)	Name statistical software and package(s), including version and settings used	9

		b)	State whether the study protocol and details were pre-registered (as well as when and where)	NA
	**RESULTS**			
10	**Descriptive data**			
		a)	Report the numbers of individuals at each stage of included studies and reasons for exclusion. Consider use of a flow diagram	NA
		b)	Report summary statistics for phenotypic exposure(s), outcome(s), and other relevant variables (e.g. means, SDs, proportions)	NA
		c)	If the data sources include meta-analyses of previous studies, provide the assessments of heterogeneity across these studies	NA
		d)	For two-sample MR:<br>i. Provide justification of the similarity of the genetic variant-exposure associations between the exposure and outcome samples	6, 7
			ii. Provide information on the number of individuals who overlap between the exposure and outcome studies	16
11	**Main results**			
		a)	Report the associations between genetic variant and exposure, and between genetic variant and outcome, preferably on an interpretable scale	Table S20-S21
		b)	Report MR estimates of the relationship between exposure and outcome, and the measures of uncertainty from the MR analysis, on an interpretable scale, such as odds ratio or relative risk per SD difference	12

		c)	If relevant, consider translating estimates of relative risk into absolute risk for a meaningful time period	NA
		d)	Consider plots to visualize results (e.g. forest plot, scatterplot of associations between genetic variants and outcome versus between genetic variants and exposure)	Figure 3 and Figure S4
12	**Assessment of assumptions**			
		a)	Report the assessment of the validity of the assumptions	12, 13
		b)	Report any additional statistics (e.g., assessments of heterogeneity across genetic variants, such as I^2, Q statistic or E-value)	NA
13	**Sensitivity analyses and additional analyses**			
		a)	Report any sensitivity analyses to assess the robustness of the main results to violations of the assumptions	12, 13
		b)	Report results from other sensitivity analyses or additional analyses	13
		c)	Report any assessment of direction of causal relationship (e.g., bidirectional MR)	13
		d)	When relevant, report and compare with estimates from non-MR analyses	NA
		e)	Consider additional plots to visualize results (e.g., leave-one-out analyses)	Figure S3
	**DISCUSSION**			
14	**Key results**		Summarize key results with reference to study objectives	15
15	**Limitations**		Discuss limitations of the study, taking into account the validity of the IV assumptions, other sources of potential bias, and imprecision. Discuss both direction and magnitude of any potential bias and any efforts to address them	16

16	**Interpretation**		
		a) Meaning: Give a cautious overall interpretation of results in the context of their limitations and in comparison with other studies	15, 16
		b) Mechanism: Discuss underlying biological mechanisms that could drive a potential causal relationship between the investigated exposure and the outcome, and whether the gene-environment equivalence assumption is reasonable. Use causal language carefully, clarifying that IV estimates may provide causal effects only under certain assumptions	16
		c) Clinical relevance: Discuss whether the results have clinical or public policy relevance, and to what extent they inform effect sizes of possible interventions	16
17	**Generalizability**	Discuss the generalizability of the study results (a) to other populations, (b) across other exposure periods/timings, and (c) across other levels of exposure	16, 17
	**OTHER INFORMATION**		
18	**Funding**	Describe sources of funding and the role of funders in the present study and, if applicable, sources of funding for the databases and original study or studies on which the present study is based	2
19	**Data and data sharing**	Provide the data used to perform all analyses or report where and how the data can be accessed, and reference these sources in the article. Provide the statistical code needed to reproduce the results in the article, or report whether the code is publicly accessible and if so, where	18
20	**Conflicts of Interest**	All authors should declare all potential conflicts of interest	18

This checklist is copyrighted by the Equator Network under the Creative Commons Attribution 3.0 Unported (CC BY 3.0) license.

1. Skrivankova VW, Richmond RC, Woolf BAR, Yarmolinsky J, Davies NM, Swanson SA, et al. Strengthening the Reporting of Observational Studies in Epidemiology using Mendelian Randomization (STROBE-MR) Statement. JAMA. 2021;under review.

2. Skrivankova VW, Richmond RC, Woolf BAR, Davies NM, Swanson SA, VanderWeele TJ, et al. Strengthening the Reporting of Observational Studies in Epidemiology using Mendelian Randomisation (STROBE-MR): Explanation and Elaboration. BMJ. 2021;375:n2233.
